# Self-Reported Dental Fear among Dental Students and Their Patients

**DOI:** 10.3390/ijerph9010044

**Published:** 2011-12-29

**Authors:** Junia Serra-Negra, Saul M. Paiva, Mauricio Oliveira, Efigenia Ferreira, Fernanda Freire-Maia, Isabela Pordeus

**Affiliations:** 1 Department of Pediatric Dentistry and Orthodontics, Universidade Federal de Minas Gerais, Avenida Presidente Antonio Carlos, 6627-Pampulha, Minas Gerais 31270-901, Brazil; Email: smpaiva@uol.com.br (S.M.P.); mauricioliveira14@yahoo.com.br (M.O.); fernandabartolomeo@gmail.com (F.F.-M.); isabela@netuno.lcc.ufmg.br (I.P.); 2 Department of Oral Public Health, Universidade Federal de Minas Gerais, Avenida Presidente Antonio Carlos, 6627-Pampulha, Minas Gerais 31270-901, Brazil; Email: efigeniaf@gmail.com

**Keywords:** behavior, fear, pain, undergraduate student, dentistry, epidemiology, health, patients

## Abstract

The aim of the present study was to compare self-reported dental fear among dental students and patients at a School of Dentistry in Belo Horizonte, Brazil. Eighty students ranging in age from 20 to 29 years and 80 patients ranging in age from 18 to 65 years participated in the study. A self-administered pre-tested questionnaire consisting of 13 items was used for data acquisition. The city of Belo Horizonte Social Vulnerability Index (SVI) was employed for socioeconomic classification. The chi-square test and binary and multinomial logistic regression were employed in the statistical analysis, with the significance level set at 0.05. The majority of dental students (76.5%) sought the dentist for the first time for a routine exam, while patients (77.3%) mostly sought a dentist for the treatment of dental pain. Dental fear was more prevalent among the patients (72.5%) than the students (27.5%). A total of 47.1% of the students and 52.9% of the patients reported having had negative dental experiences in childhood. The logistic model revealed an association between dental fear and a pain-related experience (OR: 1.8; 95%CI: 1.3–2.6). Patients were more prone to dental fear (OR: 2.2; 95%CI: 1.0–5.0). Although at different percentages, both students and patients experienced dental fear. Current patient with previous experience of dental pain had more dental fear.

## 1. Introduction

Fear is described as an apprehensive and uncomfortable feeling [1]. Dental fear refers to the fear of dentistry. It is not unusual to detect dental fear among patients, which may constitute a barrier to seeking dental care [2,3]. A large number of patients only seek a dentist when they have a toothache [2]. Some individuals experience fear due to certain stimuli involved in dental treatment. The sight of a needle, feeling of the injection and noise of the drill and other dental instruments trigger emotional sensations of discomfort [2]. An investigation into dental fear involving 169 Japanese dental and nursing students between 18 and 37 years of age found that the most fear-provoking items were the sight and feeling of the needle and the sound and feeling of the drill [3]. This uncomfortable sensation often leads patients to putting off dental care [4,5,6]. 

Fear is observed in children, adults and the elderly [3]. The first visit to the dental office is an important milestone in a child’s life and the timely visit should be an essential part of the child’s general health care [7,8,9]. Some countries have social difficulties regarding access to oral health services among underprivileged populations [10,11]. Both those who receive care and those who offer oral healthcare services may exhibit dental fear, which can adversely affect health, as such fear is often a reason for the postponement or cancellation of appointments [4,5,6].

A study involving 1,600 Saudi Arabian students found that 22% displayed a high degree of dental fear [12]. A study carried out in Pakistan assessed discomfort with regard to dental treatment among 503 university students and found that 21.6% of the men and 24.0% of the women reported negative sensations in relation to dental treatment [13]. A British study assessed the fear of dental pain among 1,800 students and found that 17% reported dental fear and 73% reported that oral health status affects quality of life [14]. A study carried out in Jordan with 600 undergraduate students of medicine, engineering and dentistry found that dental students had lowest percentage of dental anxiety (11.22%) and medical students had the highest percentage (13.58%) [15]. In a study carried out in Brazil 13% of university students reported a high degree of dental fear [16].

The nature of the dentist/patient relationship is of utmost importance. Patients tend to be calmer when the dentist projects confidence [17]. Dental students preparing for a professional career must not only learn dental techniques, but also how to deal with patient fear and anxiety in children, adults and the elderly [3,15]. The aim of the present study was to compare self-reported dental fear in a group of dental students who offer dental services and a group of patients who receive care from dental students to determine whether the presence of dental fear affects the behavior of both.

## 2. Methods

### 2.1. Subjects

Eighty patients and 80 dental students (a total of 160 participants) at the undergraduate course of the School of Dentistry, *Universidade Federal de Minas Gerais* (UFMG) in the city of Belo Horizonte, Brazil, participated in the present study. Among the students age ranged from 20 to 29 years (mean: 22.2) and from 18 to 65 years among the patients (mean: 40.5). The methodology was tested in a pilot study on a sample of 40 patients and 40 students enrolled in the final semester of dental course. These individuals did not take part in the main sample. The results of this pilot study indicated the need for modifications to improve the quality of the data acquisition. A final modification of the questionnaire was carried out based on the questions and suggestions that arose during the pilot study. Test-retest reliability was assessed using 20 randomly selected participants (10 students and 10 patients), with intra-class correlation values for final model of the questionnaire ranging from 0.72 to 0.85 for the students and 0.70 to 0.80 for the patients.

The current curriculum of the UFMG undergraduate dental program consists of eight semesters. One hundred twenty students are enrolled annually in this dental school. The target sample population consisted of 99 undergraduate dental students in the 4th and 8th semesters. The 4th semester of was chosen due to the fact that it is the first semester with a clinical component and the 8th semester was chosen due to the fact that it is the last semester with a clinical component and finalizes the theoretical component of the curriculum. For the main study, an informed consent form was distributed to the target population (99 students) during lecture classes. Student participation in the study was voluntary. The questionnaire was distributed to the students who agreed to participate. There was a return of 80 questionnaires, totaling an 80.8% response rate.

Each dental student sees a group of patients pertaining to different dental disciplines. At the dental school, each student receives an average of two patients per discipline per semester. As students have an average of five disciplines per semester, each student sees a total of 10 patients per semester. The list of all patients was requested from the institution with the name of the student responsible for each patient. In this manner, one patient was randomly selected from each student. The patients were contacted in the waiting room of the UFMG dental clinics. As there were 80 students, the sample of patients was also 80. The patients were unaware that the students who had treated them were also participating in the study and the students were not informed that their patients were participating. This information was maintained confidential to avoid possible interferences in the responses. 

### 2.2. Questionnaire

The decision was made to use the same questionnaire for both students and patients. This concern was based on the fact that the two groups had different socio-cultural characteristics. An easy-to-understand language was used to ensure that both two groups clearly and objectively understood what was being asked. 

The self-administered questionnaire consisted of 13 questions (3 open-ended questions and 10 multiple choice items). The same pre-tested questionnaire was self-administered to the students and patients and was made up of questions on current age, gender, place of residence, category (student or patient), age at first visit to the dentist, presence or absence of dental fear, reason for first visit to the dentist, negative experiences at the dental office in childhood, preference for the dentist’s gender and situations and instruments of discomfort during dental treatment. The answers for the multiple choice items were “yes-or-no”. The questionnaire is reproduced below:

1. Are you a patient or dental student?
2. How old are you?
3. What is the address of your residence?
4. Do you remember how old you were the first time you visited the dentist? How old?
5. Do you remember the reason for your first visit to the dentist?
6. How many times a year do you go to the dentist? For what reason do you seek the dentist?
7. Have you ever cancelled a dental appointment because of fear?
8. Do you remember any bad dental experience at the dentist in childhood?
9. When you go to the dentist, do you perceive any of the following reactions? You can mark more than one option:
  ( ) Your breathing becomes faster.
  ( ) Your hands become sweaty.
  ( ) Your heart beats faster.
  ( ) You sleep poorly the night before the appointment.
10. When you schedule an appointment with the dentist, do you choose the gender of the dentist who will treat you?
11. If you answered positively to the previous question, what gender do you prefer for the dentist?
12. Are there any situations or instruments that bother you during dental care? Which?
13. Do you have dental fear?

Participants who gave at least two affirmative responses to Question 9 or responded yes to Question 13 were considered as having dental fear. Affirmative answers on Question 9 were based on physiological reactions to fear [18].

### 2.3. Social Class Classification

The Social Vulnerability Index (SVI) was employed for socioeconomic classification [19]. This area-based index measures social exclusion in the city of Belo Horizonte and is based on cultural, social and demographic contexts. Area-based measures of deprivation offer a number of advantages, which accounts for their increasing importance in the improvement of healthcare planning and policies. Socially homogenous neighborhoods do not commonly differ in terms of social welfare, attitudes or behavior [11]. The SVI encompasses over twenty variables that quantify the population’s access to housing, schooling, income, jobs, legal assistance, health and nutrition. Thus, the SVI measures social access and determines to what extent the population of each region of the city is vulnerable to social exclusion. In the present study, the city hall database on SVI scores for each region was used based on the address of each participant [18]. This index is classified in five categories. Class I represents regions of the greatest vulnerability and Class IV represents the least vulnerability. For statistical purposes, the SVI was dichotomized as high (Classes I, II and III) and low (Classes IV and V) degrees of social vulnerability. 

### 2.4. Statistical Analysis

The normality of data distribution was determined using the Kolmogorov-Smirnov test, which did not confirm the assumption of normality. Associations between the outcome variable (dental fear) and exogenous variables were determined using the non-parametric chi-square test. The significance level was set at α = 0.05. To estimate the probability of the occurrence of dental fear, the data were analyzed using binary and multinomial stepwise logistic regression. Multinomial logistic model was used because of the interest in studying the two groups: comparing both dental students and their patients with the dental fear. The order in which variables were input for multinomial logistic model was guided by the theoretical model and by the statistical significance of the association given by univariate analysis with p < 0.05. Dental fear was the outcome variable. All exogenous variables were included in the multinomial logistic model. The UFMG Human Research Ethics Committee approved the protocol for this study.

## 3. Results

### 3.1. Sample Characterization and Prevalence of Dental Fear

Table 1 displays the values of association of behavior between students and patients with all variables studied after chi-square analysis. Fifty-two (32.5%) of the participants were male and 108 (67.5%) were female. The majority of students (81.9%) lived in areas of lesser social vulnerability, whereas the majority of patients (69.7%) lived in areas of greater social vulnerability. The majority of dental students (76.5%) sought the dentist for the first time in childhood for a routine check-up, whereas the majority of patients (77.3%) sought the dentist for the first time in childhood due to a toothache. The average age at the first dental visit was six years among the students and 13 years among the patients. Twelve percent of the participants could not recall their age upon their first dental visit. Dental fear was more prevalent among the patients (72.5%) and among women (51.9%), but this association was non-significant. Dental fear was very similar among the students in the 4th semester (26.8%) and 8th semester (27.4%) of the dental course (p = 0.401). A total of 47.1% of the students and 52.9% of the patients reported having negative experiences at the dental office in childhood. The variables regarding treatment that achieved the highest fear index values were the sight of the needle, the sensation of injection during anesthesia (48.9%) and the sound and sensation of the drill on the teeth (27.6%). Both patients and students alike reported not having a preference with regard to the gender of the dentist.

**Table 1:** Association of behavior between students and patients with variables.

Variables	Student	Patient	p-value
	N (%)	N (%)	
Dental fear			
No	69 (57.5)	51 (42.5)	<0.001 *
Yes	11 (27.5)	29 (72.5)	
Type of dental experience			
Negative	64 (47.1)	72 (52.9)	0.060
Positive	16 (84.2)	08 (15.8)	
Reason for 1st visit			
Routine check-up	62 (76.5)	19 (23.5)	<0.001 *
Dental Pain	18 (22.7)	61 (77.3)	
Social Vulnerability Index (SVI)			
Low	50 (81.9)	11 (18.1)	<0.001 *
High	30 (30.3)	69 (69.7)	
Gender of participant			
Male	28 (53.8)	24 (46.2)	0.306
Female	52 (48.1)	56 (51.9)	
Gender preference for dentist			
No	45 (51.1)	43 (48.9)	0.437
Yes	35 (48.6)	37 (51.4)	

Notes: N = number, p-value = probability value, “%” = percent and * chi-square test at a 5% level.

Table 2 displays the values of the multinomial logistic regression model regarding presence of dental fear and other exogenous variables studied. Dental fear was the outcome variable. All exogenous variables were included in the multinomial logistic model. The adjusted logistic model revealed an association between dental fear and the experience of pain (OR: 1.8; 95%CI: 1.3–2.6) (p < 0.001) and that the patients were more prone to dental fear than the students (OR: 2.2; 95%CI: 1.0–5.0) (p < 0.001). The Social Vulnerability Index (SVI) was not significant (p = 0.054). That means that this model did not find significant predictor that might explain the association with dental fear, “social vulnerability”, “type of dental experience”, “gender” and “gender preference for dentist”. Probably, there was interaction between the variables. The majority of patients lived in areas of greater social vulnerability and the majority of participants were women. Perhaps this is a justification by this interaction. The adjusted logistic model determined that dental patients with previous dental pain experience (OR = 1.8) are twice as likely to have dental fear (OR = 2.2) than those who not reported dental fear.

**Table 2:** Multinomial logistic regressionmodel regarding presence of dental fear and other exogenous variables.

Variables	Non-adjusted OR (95%CI)	p-value	Adjusted OR (95%CI)	p-value
Participants				
Students	1		1	
Patients	2.2 (1.0–5.0)	0.001 *	2.1 (0.9–4.7)	<0.001 *
Reason for 1st visit				
Routine check-up	1		1	
Dental Pain	1.9 (1.3–2.6)	0.001 *	1.8 (1.3–2.6)	<0.001 *
Social Vulnerability Index(SVI)				
Low	1		1	
High	2.0 (1.2–3.1)	0.002 *	1.6 (0.9–2.6)	0.054
Type of dental experience				
Negative	1		1	
Positive	0.7 (0.54–1.03)	0.060	0.7 (0.54–1.05)	0.098
Gender of participant				
Male	1		1	1
Female	0.6 (0.26–1.73)	0.432	0.9 (0.65–1.27)	0.581
Gender preference for dentist				
No	1		1	
Yes	0.9 (0.98–1.00)	0.429	0.7 (0.54–1.05)	0.580

Notes: OR = odds ratio, 95%CI = 95% confidence interval, p-value = probability value and * chi-square test at a 5% level.

## 4. Discussion

The purpose of the present study was to identify and compare dental fear among students and patients at the main dental school in the city of Belo Horizonte, Brazil. Dental fear was found in both groups of participants, but with a higher prevalence among the patients. Social factors may explain this behavior, as the majority of patients pertained to the lower classes, which tend to have more difficulties regarding access to dental services in comparison to more privileged classes [11,20]. Most patients were from areas of high vulnerability to social exclusion (69.7%) and low socioeconomic status. 

The institution offers free care provided by undergraduate students. However, due to the considerable demand, difficulties are encountered in obtaining dental services. Therefore, patients often arrive with serious treatment needs and a greater chance of experiencing pain, which increases the chances of the patient becoming more anxious [8,9]. This is the vicious circle of fear that affects oral health [4,5,6].

The same reasoning can be employed when measuring age upon the first dental visit. The average age reported by patients was 13. A study involving Indian children found a greater prevalence of older children upon their first visit to the dentist (59%) in comparison to younger children (23%) [7]. In the present study, there was demand for the dentist when patients were already in their teens and therefore in the permanent dentition phase. Brazil is a developing country with social difficulties in access to oral health services among underprivileged populations [10]. Dental fear may adversely affect health as such fear is often a reason for the postponement or cancellation of appointments [4,5,6].

In the present study, no significant association was found between dental fear and gender, which is in disagreement with the findings described in previous studies [12,13,15,16]. Among the stimuli studied, those that achieved the highest fear index values were the sight of the needle, the sensation of injection during anesthesia (48.9%) and the sound and sensation of the drill on the teeth (27.6%). The findings corroborate those reported by Yoshida *et al*. (2009) [3]. 

While assessment tools for the measurement of dental fear have been validated for use in Brazil, the decision was made not to employ such tools, but rather to draft an original questionnaire in an easy-to-understand language to ensure that both two groups clearly and objectively understood what was being asked. However, the concern regarding the sufficient understanding of all participants remains as the two groups had different socio-cultural characteristics. This methodology was employed despite knowing that it would constitute a limitation in the present study. Test-retest reliability of the questionnaire was carried out, with intra-class correlation coefficients ranging from 0.72 to 0.85 for dental students and 0.70 to 0.80 for patients. 

A total of 27.5% of the dental students reported having a fear of dental treatment when they themselves are patients. In a study carried out in Jordan, 11.22% of dental students reported having dental fear [15]. In a Brazilian study, 13% of undergraduate psychology students exhibited a high degree of dental fear [16]. These differences are likely due to the use of different assessment tools for measuring dental fear. Another characteristic of the student group in the present study was that 47.1% reported having a traumatic dental experience in childhood. These data lead to reflections with regard to teaching dentistry and the profile of students. In a study carried out in Ireland, undergraduate dental students were asked to score the influence of certain factors on their decision to choose dentistry as a career; the reasons cited included perceived ease of employment, being self-employed, working regular hours, good income and the opportunity to help people [21]. In order for dental students to be able to help people, they must receive secure support in both theory and practice during their education [20,21,22,23,24]. If a student feels insecure while performing treatment, he/she passes this insecurity on to the patient, thereby reinforcing dental fear [17,21,23,25].

It should be pointed out that a cross-sectional study design with retrospective data collected by means of self-administered questionnaires could lead to the occurrence of memory bias as well as reverse causality, which suggests the need for further research using different study designs. Before considering whether exposure may lead to a given outcome, one must consider whether the outcome may have caused the exposure [26,27]. 

A total of 72.5% of the patients reported fear of the dentist. The majority of patients were actually contacted in the waiting room for dental surgery and periodontal treatment. Surgical treatment traditionally generates greater anxiety in comparison to less invasive procedures [1]. In any case, fearful patients report a need to feel secure about the professional who is treating them [17,26,27,28], but also report the expectation of experiencing pain when coming to the dental clinic, which further reinforces the fact that pain is a risk factor for dental fear [8].

It is important to consider the limitations of the present study. This is a cross-sectional study and it is important to note that perceptions of dental fear can change over time. Cross-sectional studies are carried out at a single point in time. Thus, the associations identified in such studies should not be considered a causal relationship. However, there is a lack of studies that assess the experience of dental pain among dental patients and dental students.

## 5. Conclusions

Dental fear was found in both dental students and patients, with a higher prevalence among patients. Our findings suggest that current patients with previous experience with dental pain had more dental fear when compared with participants that no reported dental fear. Teaching and health policies should be encouraged in order to favor access to preventive dental services among the majority of the population in an attempt to reduce exposure to pain and diminish fear related to dental care. The nature of the dentist/patient relationship is of utmost importance. Dental students offering dental services should project a sense of security to the dental patient and the patient receiving dental care should feel confident in the dentist who is treating him/her.
